# Knowledge, Attitude, and Practice Survey of COVID-19 Among Healthcare Students During the COVID-19 Outbreak in China: An Online Cross-Sectional Survey

**DOI:** 10.3389/fpubh.2021.742314

**Published:** 2021-10-07

**Authors:** Juxia Zhang, Yuhuan Yin, Judith Dean, Xiaoli Zhang, Yiyin Zhang, Jiancheng Wang, Yinping Zhang

**Affiliations:** ^1^Clinical Educational Department, Gansu Provincial Hospital, Lanzhou, China; ^2^School of Nursing, Gansu University of Chinese Medicine, Lanzhou, China; ^3^School of Public Health, The University of Queensland, Herston, QLD, Australia; ^4^Geriatrics Department, Gansu Provincial Hospital, Lanzhou, China; ^5^School of Nursing, Xi'an Jiaotong University Health Science Center, Xi'an, China

**Keywords:** knowledge, attitude, practice, COVID-19, China, health care

## Abstract

**Background:** The ongoing coronavirus disease (COVID-19) outbreak has placed the healthcare system and student training under considerable pressure. However, the plights of healthcare students in the COVID-19 period have drawn limited attention in China.

**Methods:** A cross-sectional on-line survey was undertaken between January and March 2020 to explore the COVID-19 knowledge, attitude, and practice (KAP) survey among Chinese healthcare students. Demographic information and data on KAP were obtained using a self-reported questionnaire. The percentage KAP scores were categorized as good or poor. Independent predictors of good knowledge of COVID-19 were ascertained to use a logistic regression model.

**Results:** Of the 1,595 participants, 85.9% (1,370) were women, 53.4% were junior college students, 65.8% majoring in nursing, and 29.8% had received training on COVID-19. The overall median percentage for good KAP was 51.6% with knowledge of 28.3%, attitude 67.8%, and practice 58.6%, respectively. Independent predictors of good knowledge of COVID-19 were being students ≥25 (95% CI = 0.27–0.93, *P* = 0.02), those taking bachelor degrees (95% CI = 1.17–2.07, *P* = 0.00), and those having participated in COVID-19 treatment training.

**Conclusions:** The result of this study revealed suboptimal COVID-19-related KAP among healthcare students in China. To effectively control future outbreaks of COVID-19, there is a need to implement public sensitization programs to improve the understanding of COVID-19 and address COVID-19-related myths and misconceptions, especially among healthcare students.

## Introduction

On December 12, 2019, several novel coronavirus pneumonia cases were detected in Wuhan, which quickly spread globally in a short period (1, 2). With the rapid, sharp increase in the reported cases worldwide, frontline healthcare workers (HCWs) have been disproportionately affected and at high risk of infection (3, 4). This risk has been further exacerbated by the lack of personal protective equipment (PPE) (5). The ever-increasing number of confirmed and suspected cases with coronavirus disease (COVID-19), overwhelming workload, depletion of PPE, and lack of specific drugs has also had a significant negative impact on the mental health and well-being of HCWs with anxiety, depression, and psychological crisis being reported among the global healthcare workforce (6). To combat the disease, the WHO has emphasized the importance of accurate clear COVID-19-related health information as a means of dispelling misconceptions, mitigating fear, preventing discrimination, and ultimately eliminating future outbreaks of COVID-19 (2). The Center for Disease Control and Prevention standard infection control guidelines details guidelines for practices that are regularly updated to protect not only the patients but also the HCW providing care to those infected (3, 7). These measures include hand hygiene, use of PPE, and correct use of medical masks and more. To effectively reduce the risk of COVID-19 transmission in healthcare institutions, HCWs need to strictly implement standardized preventive measures and strengthen protective measures (1), to do this HCW and healthcare students need the knowledge, skill, and equipment to adhere to these standardized measures.

The risk for healthcare students in China has been mitigated by the Chinese Ministry of Education prohibiting them from returning to their medical schools for studies or undertaking clinical work in hospital settings until further notification (8). To maintain these strong restrictions, medical schools have adapted to online teaching and learning platforms (9). Independent on-line study has become an important skill for healthcare students worldwide (10). While this may have reduced the risk of students for exposure to COVID-19, but students, especially those living in rural areas with underdeveloped networks and poor hardware facilities, may find it difficult to meet the requirements of online learning (8). Unease and unfamiliarity with online teaching methods among students (11) may also affect the mental health and well-being of students due to concerns about their ability to successfully complete the academic year (12). Progression, mental health, and well-being of the students may be further affected by COVID-19-related social isolation and future uncertainties (12), along with the dangers associated with exposure to infectious diseases (8), manifesting in depression, anxiety, and suicidal ideations (13, 14).

Healthcare workforce shortages have resulted in healthcare students being strongly encouraged to volunteer (10). Several studies have found students to be highly motivated to volunteer in medical-related work (12), such as being community volunteers (15), ventilator therapy assistants, and nursing assistants (16, 17) as this may provide an opportunity for them to continue their education efficiently by returning to clinical rotations. Moreover, healthcare students have taken up roles in educating the public and increasing their awareness of the COVID-19 pandemic (18). However, for students to effectively practice in these roles, they need to be aware of all government advisories, guidelines, and standardized prevention and protective measure for their safety and well-being. Despite efforts, investigations indicate that there are still students in pharmacy, dental, medical, and public health areas neglecting the benefit of social distancing, staying at home, and the use of other preventive measures, such as using sanitizer and masks due to attitudinal and behavioral issues (19–22). Lessons learned from Ebola and severe acute respiratory syndrome (SARS) indicated that public perceptions, attitudes, and behaviors play an important role in the effective control of epidemic disease (23–25). Early prevention is an important strategy in the global fight against COVID-19 (26). However, inadequate knowledge of COVID-19 and standardized preventative measures may influence attitudes and practices of healthcare students and directly increase their risk of infection (12).

Healthcare students are the future of a sustainable health system. COVID-19 has placed the health system and student preparation under severe pressure. However, the plights of healthcare students in the COVID-19 period have drawn limited attention in China. So far, there has been no comprehensive investigation on the KAP of COVID-19 among healthcare students with different specialties, such as nursing, clinical medicine, clinical laboratory, radiology, midwifery, and psychology. Thus, this study has two aims (1) to explore the current situation of KAP of the Chinese healthcare students on COVID-19 and (2) to investigate the factors affecting KAP, to provide a more accurate reference for the basic training of healthcare students in the future.

## Materials and Methods

### Study Design and Participants

A cross-sectional survey was conducted by using an online self-administered questionnaire. The target population was a convenience sample of students enrolled, at any year level, in an undergraduate healthcare profession program majoring in nine specialty areas (clinical medicine, nursing, clinical laboratory, radiology, midwifery, traditional Chinese medicine, rehabilitation medicine, and psychology, pharmaceutics) at 26 universities or junior colleges in 10 provinces, who had the capacity to provide voluntary informed consent. To assist with the snowball sampling approach applied, we recruited 10 collaborators who are the managers of students. The collaborators were given an online orientation session about the nature of the study and the data collection strategy. The 10 collaborators invited an initial group of eligible students to participate: the first set of invitees then forwarded the invitations to their contacts whom they considered suitable, and this second set forwarded the invitation in the same way.

### Sample Size Calculation

The sample size was calculated by using the formula N = Z^2^**·***P*(1–*P*)/E^2^ (27), based on the following assumption: the proportion of good knowledge 50%, level of confidence 99%, and precision 5%; then, the sample size was increased by 10% to overcome non-response. The minimum sample size was 733.

### Instruments

Demographic data include gender, age, home location, nationality, religion, grade, level of study, specialty, whether or not having received training on COVID-19, and whether or not having participated in volunteer. The KAP questionnaire was developed based on the relevant WHO guidelines, training workshop materials on emerging respiratory diseases, such as COVID-19, in addition to the previously published surveys of other pandemics awareness (24). Subsequently, 15 experts from the departments of Public Health and Internal Medicine (division of infectious diseases) at Gansu Provincial Hospital (Gansu, China) were consulted, and the investigators modified the questionnaire accordingly. The content validity index (CVI) and content validity ratio (CVR) of experts were calculated. A preliminary pilot experiment was conducted on 30 healthcare students who interned in Gansu Provincial Hospital, and a predictive test questionnaire was formed. To verify the reliability and validity of the questionnaire, 325 health students in Gansu Provincial Hospital were selected for a formal investigation. The official questionnaire was formed after the data of valid questionnaires were analyzed. The reliability and validity of the official version of the questionnaire are tested to form the final version of the questionnaire. The questionnaire included knowledge about COVID-19 and consisted of 12 questions about COVID-19 mode of transmission, vulnerable person for infection, symptoms, and prevention measures. Correct answers scored 1 and incorrect answers scored 0. Attitudes toward COVID-19 consisted of 10 questions assessing optimism about the current situation, responsible public health attitudes, stigma against symptomatic individuals and healthcare professionals, and whether the participant like to be volunteered for the vaccine. Each item was answered with “yes” (0 point), “no” (1 points), or “not sure” (0 points). The practice section included 14 questions describing different practices regarding coughing and sneezing, hand washing, wearing masks, and contact with people. The available answers to each question were “yes” (1 point), “no” (0 point), or “not sure” (0 point).

### Institutional Review Board Approval

The study was conducted in accordance with the Declaration of Helsinki. Because the university was on lockdown during the outbreak, the research proposal was approved by the research committee of Gansu Provincial Hospital. The questionnaire contains informed consent, which explains the purpose of the study, nature of the survey, objectives of the study, voluntary participation, declaration of confidentiality, anonymity, and other information.

### Data Collection

Data were collected from January to March 2020. Due to public health directives to reduce the risk of COVID-19 transmission, the survey was conducted online. The investigators converted the questionnaire into the Questionnaire Star Platform, the most popular communication and social platform in China (28), and then distributed online version by means of the We Chat platform to the first set of invitees. All items were required to be filled in, and the same IP address could only be answered once. Responses from participants across all items were summed and transformed to KAP scores for each component. The central investigator had access to all responses. After the data collection, we used Microsoft Excel for data cleaning.

### Statistical Analysis

SPSS21.0 and Amos 21.0 were used to analyze the reliability and validity of the questionnaire. Factor analysis was used to test the structure validity. CVI and CVR were used to analyze the content validity. The internal correlation was tested by the Pearson correlation coefficient. Cronbach's coefficient and mean item correlation coefficient (MIIC) were used to test the reliability of the questionnaire.

Based on the distribution of respondent scores, cutoff scores for knowledge ≥10, attitude ≥6, and practice ≥8 were used to classify the KAP score into good and poor (24). Chi-squared test, independent sample test, and one-way analysis of variance were used to compare differences in knowledge, attitude, and practice of medical students by demographic characteristics. Categorical variables were measured as percentages while continuous variables were expressed as mean ± SD. Using the appropriate KAP scores as cutoff points based on performance, we categorized the KAP scores into two groups, namely, good KAP and poor KAP. Differences in study variables according to good or poor knowledge of COVID-19 were explored using *t*-test for quantitative variables and Fisher's test for categorical variables. Predictors of good vs. poor knowledge were determined with the results expressed as odds ratio (OR) and 95% CI. Variates associated with good knowledge were imputed in a Logistic Regression model to determine independent predictors of good knowledge and practice of COVID-19. The model was constructed using a backward stepwise approach. *P* < 0.05 was considered to indicate significance in all tests; statistical analysis was carried out using SPSS version 21.0.

## Results

### Reliability and Validity of the Questionnaire

The results showed that CVI = 0.923, the range of CVI of each item was 0.857–1.00, CVR = 0.846. The Pearson correlation coefficient between each factor was 0.402–0.481, and the correlation coefficient between each factor and the total score of the scale was 0.613–0.774.

The total Cronbach's coefficient of the questionnaire was 0.952. Cronbach's instrument coefficients of each factor were 0.910, 0.885, and 0.809, respectively. The MIIC value of the questionnaire was 0.293, and the MIIC values of each factor were 0.666, 0.629, and 0.642.

### Study Population and Demographic Characteristics

In total 1,595 valid questionnaires from 26 universities were included. The demographic characteristics of the studied population are shown in Table 1. Of the 1,595 students, 1,370 (85.9%) were women, 1,071 (67.1%) of the Han nationality, and 1,067 (66.9%) were free thinkers. The respondents lived in 12 provinces and cities, such as Gansu, Hubei, Hunan, Henan, Hebei, Ningxia, Beijing, and 1,058 (66.3%) resided in rural areas. A third (490, 30.7%) had the experience of clinical practice, 851 (53.4%) were junior college students, and 1,049 (65.8%) were majoring in nursing. Just under a third (475, 29.8%) had received training on COVID-19 and 224 (14.0%) had participated in local volunteer activities.

**Table 1:** Demographics of included students (N = 1,595).

**Characteristics**	**Total N**	**%**
**Gender**
Female	1,370	85.9
Male	225	14.1
**Age**
≤ 20	484	30.3
21-24	1,038	65.1
≥25	73	4.6
**Home location**
Rural	1,058	66.3
Urban	537	33.7
**Nationality**
Han	1,070	67.1
Muslim	464	464 (29.1)
Tibetan	42	2.6
Others	19	1.1
**Religion**
Buddhism	60	3.8
Islam	442	27.7
Christianity	7	0.4
Atheist	1,067	66.9
Others	19	1.2
**Grade**
First year	311	19.5
Second year	408	25.6
Third year	386	24.2
Fourth year	490	30.7
**Level of education**
Bachelors	744	46.6
Junior college	851	53.4
**Specialty**
Nursing	1,049	65.8
Clinical medicine	234	14.7
Clinical laboratory	91	5.7
Radiology	50	3.1
Midwives	36	2.3
Traditional Chinese Medicine	78	4.9
Stomatology	18	1.1
Rehabilitation medicine	11	0.7
Psychology	8	0.5
Pharmacology	20	1.3
**Received training on COVID-19**
Yes	475	29.8
**Participated in local volunteer activities**
Yes	224	14

### Knowledge, Attitude, and Practice of COVID-19

Percentages of scores for KAP were obtained. The differences in the study variables in relation to the KAP of COVID-19 are presented in Table 2.

**Table 2:** Differences in knowledge, discrimination, and practice by demographics.

**Characteristics**	**Knowledge^a^**		**Discrimination^b^**		**Practice^c^**	
	**Good (%)**	**X^**2**^ (*P*)**	**Number (%)**	**X^**2**^ (*P*)**	**Good (%)**	**X^**2**^ (*P*)**
Overall	451 (28.3)		1,083 (32.2)		934 (58.6)	
Gender		0.29 (0.57)		4.11 (0.12)		2.69 (0.10)
Female	384 (28.03)		522 (38.10)		791 (57.74)	
Male	67 (29.78)		71 (31.56)		143 (63.56)	
Age		12.71 (**0.002**)		12.71 (**0.002**)		2.08 (0.35)
≤ 20	113 (23.35)		113 (23.35)		282 (58.26)	
21–24	308 (29.67)		308 (29.67)		615 (59.25)	
≥25	30 (41.10)		30 (41.10)		37 (50.68)	
Home location		0.23 (0.63)		1.28 (0.52)		3.52 (0.06)
Rural	295 (27.88)		383 (36.20)		637 (60.21)	
Urban	156 (29.05)		210 (39.11)		297 (55.31)	
Nationality		2.78 (0.42)		2.78 (0.42)		3.55 (0.31)
Han	313 (29.25)		313 (29.25)		620 (57.94)	
Muslim	125 (26.94)		125 (26.94)		275 (59.27)	
Tibetan	10 (23.81)		10 (23.81)		24 (57.14)	
Others	3 (15.79)		3 (15.79)		15 (78.95)	
Religion		5.81 (0.21)		4.38 (0.82)		4.91 (0.29)
Buddhism	11 (18.33)		23 (38.33)		38 (63.33)	
Islam	121 (27.38)		154 (34.84)		261 (59.05)	
Christianity	3 (42.86)		1 (14.29)		6 (85.71)	
Atheist	308 (28.87)		406 (38.05)		621 (58.20)	
Others	8 (42.11)		9 (47.37)		8 (42.11)	
Grade		6.15 (0.105)		14.02 (**0.02**)		4.80 (0.18)
First year	73 (23.47)		96 (30.87)		184 (59.16)	
Second year	118 (28.92)		159 (38.97)		232 (56.86)	
Third year	106 (27.46)		154 (39.90)		243 (62.95)	
Fourth year	154 (31.43)		184 (37.55)		275 (56.12)	
Level of Education		13.53 (**0.001**)		7.49 (0.11)		1.94 (0.37)
university	235 (32.50)		167 (23.10)		410 (56.71)	
Junior college	208 (24.44)		232 (27.26)		512 (60.16)	
Specialty		8.95 (0.44)		17.11 (0.51)		4.62 (0.86)
Nursing	296 (28.22)		400 (38.13)		91 (8.67)	
Clinical medicine	75 (32.05)		85 (36.32)		21 (8.97)	
Clinical laboratory	27 (29.67)		35 (38.46)		6 (6.59)	
Radiology	10 (20.00)		12 (24.00)		3 (6.00)	
Midwives	6 (16.67)		16 (44.44)		2 (5.56)	
Traditional Chinese Medicine	22 (28.21)		29 (37.18)		6 (7.69)	
Stomatology	7 (38.89)		5 (27.78)		1 (5.56)	
Rehabilitation medicine	1 (9.09)		3 (27.27)		0	
Psychology	2 (25.00)		3 (37.50)		1 (12.50)	
Pharmacology	5 (25.00)		5 (25.00)		0	
Received training on COVID-19		0.001 (1.00)		0.15 (0.92)		22.68 (**0.00**)
Yes	134 (28.21)		180 (37.89)		321 (67.58)	
Participated volunteer activities		0.14 (0.74)		0.29 (0.86)		4.70 (**0.03**)
Yes	61 (27.23)		83 (37.05)		146 (65.18)	

*Bold values are those indicating statistical significance (P < 0.05)*.

a *Total score ranged from 0 to 12. A score of ≥ 10 for good knowledge regarding COVID-19*.

b *Total score ranged from 0 to 10. A score of <6 indicates discrimination regarding COVID-19*.

c *Total score ranged from 0 to 14. A score of ≥ 8 indicates good practice regarding COVID-19*.

Total COVID-19 knowledge scores ranged from 4 to 16, with a mean score of 8.88 (SD = 0.97). Based on the classification categories of the knowledge level, a quarter of the medical students showed good knowledge (28.3%, N = 451; Table 2). The score of knowledge among healthcare students of different ages and education levels showed statistical significance (*P* < 0.05). Knowledge did not differ significantly (*P* > 0.05) with gender, home location, nationality, religion, grade, and specialty nor by whether the student had received COVID-19 training, or participated in local volunteer activities (Table 3).

**Table 3:** Variable definition for factors associated with good knowledge, attitude, and practice.

**Variable categories**	**Variable name**	**Code**
Age	X1	≥ 20 = 1, 21~24 = 2, ≥ 25 = 3
Grade	X2	First year = 1, Second years = 2, Third years = 3, Fourth years = 4
Level of education	X3	Bachelors = 1, Junior college = 2
Received training on COVID-19	X4	Yes = 1, No = 2
Participated in local volunteer activities	X5	Yes = 1, No = 2
Knowledge	Y1	Poor = 0, Good = 1
Practice	Y2	Poor = 0, Good = 1

Among the 1,595 students, 67.8% (1,081) have a good attitude, of which 79.0% (1,260) thought COVID-19 a serious disease, 82.4% (1,343) thought it could be managed properly. Moreover, 76.1% would not leave their dormitory after a roommate was infected while 62.13% (991) would return after recovery. If a friend was diagnosed with COVID-19 and cured, 62.8% of students were willing to continue their contacts. Age (X^**2**^ = 12.71, *P* = 0.002) and grade (X^2^ = 14.02, *P* = 0.02) had significant effect on attitudes toward infected roommates and friends (Table 2).

Students reported a high level of precautionary practices (M = 7.96, SD = 2.51, ranging: 0–14). According to the practice categories, most students (58.6%, *n* = 934) reported “good compliance” with 92.7% of students reporting that they always wore a mask, 52.4% were willing to volunteer for a COVID-19 vaccine, and the majority washing hands regularly with a sanitizer (57.9%) or soap (26.5%). Previous training (X^**2**^ = 22.68, *P* = 0.00), and whether or not having participated in local volunteer activities (X^**2**^ = 4.70, *P* = 0.03), significantly affect practice of students (Table 2).

### Factors Affecting KAP of Students

Factors (age, grade, education levels, and whether or not having received training on COVID-19 and participated in local volunteer activities) with statistical significance in the single-factor analysis were regarded as independent variables Xi, knowledge, and practice to COVID-19 were taken as dependent variables Y1 and Y2, respectively. The relationship between the knowledge and practice to COVID-19 and various variables was obtained, as shown in Table 3.

Logistic regression (Table 4) revealed that students ≤ 20 years old had a lower knowledge score than those ≥25 (OR = 0.50, 95% CI = 0.27–0.93, *P* = 0.02). Compared with students having a junior college degree, those with a Bachelor degree had a higher level of knowledge (OR = 1.56, 95% CI = 1.17–2.07, *P* = 0.00). Students who had participated in COVID-19 training were more actively taking prevention precautions than those who did not (OR = 1.68, 95% CI = 1.34–2.12, *P* = 0.00).

**Table 4:** Factors associated with good knowledge, attitude, and practice.

		**Good knowledge^a^**				**Good attitude^b^**				**Good practice^c^**			
**Characteristics**	**Reference**	**β**	**SE**	**P**	**OR (95%CI)**	**β**	**SE**	**P**	**OR (95%CI)**	**β**	**SE**	**P**	**OR (95%CI)**
Age	≥25			0.07				0.62				0.38	
≤ 20		−0.68	0.31	**0.02**	0.50 (0.27-0.93)	−0.42	0.47	0.37	0.65 (0.25-1.67)	0.21	0.30	0.46	1.24 (0.69-2.23)
21-24		−0.43	0.27	0.11	0.64 (0.37-1.11)	−0.39	0.41	0.33	0.67 (0.30-1.51)	0.32	0.27	0.23	1.38 (0.81-2.34)
Grade	Fourth			0.31				0.46				0.53	
First year		0.06	0.23	0.78	1.06 (0.68-1.64)	−0.45	0.38	0.22	0.63 (0.29-1.33)	0.01	0.20	0.92	1.01 (0.68-1.50)
Second year		0.31	0.18	0.09	1.37 (0.94-1.98)	0.06	0.30	0.84	1.06 (0.58-1.92)	−0.05	0.17	0.74	0.94 (0.67-1.32)
Third year		0.15	0.18	0.40	1.16 (0.81-1.65)	−0.00	0.29	0.99	0.99 (0.56-1.76)	0.15	0.16	0.34	1.17 (0.84-1.62)
Level of education	Junior college			**0.01**				0.70				0.78	
Bachelor		0.44	0.14	**0.00**	1.56 (1.17-2.07)	0.20	0.24	0.40	1.22 (0.76-1.97)	−0.09	0.13	0.49	0.91 (0.69-1.18)
Received training on COVID-19	Yes	0.07	0.12	0.57	1.07 (0.83-1.37)	0.14	0.20	0.47	1.15 (0.77-1.71)	0.52	0.11	**0.00**	1.68 (1.34-2.12)
Participated volunteer activities	Yes	−0.08	0.16	0.62	0.92 (0.66-1.27)	0.48	0.23	**0.03**	1.62 (1.03-2.56)	0.26	0.15	0.08	1.30 (0.96-1.75)

*OR, odds ratio, CI; confidence interval. P < 0.05 was considered to indicate significance. Bold values show significant differences*.

a *Total score ranged from 0 to 12. A score of ≥10 for good knowledge regarding COVID-19*.

b *Total score ranged from 0 to 10. A score of ≥6 indicates good attitude regarding COVID-19*.

c *Total score ranged from 0 to 14. A score of ≥8 indicates good practice regarding COVID-19*.

## Discussion

COVID-19 had resulted in healthcare students becoming the backup workforce for healthcare organizations; therefore, a better understanding of COVID-19 KAP of students is essential. To the knowledge of the authors, there have been no studies examining the KAP of COVID-19 among Chinese healthcare students reported. Our study will provide insight that will inform the design of future interventions that address gaps in the educational preparation of healthcare students to become safe and effective HCW in the fight against the COVID-19 outbreak.

The knowledge of the healthcare students about COVID-19 in this study is close to a study conducted in Oman (10). However, overall student knowledge was lower than scores reported from several other countries in the same area, such as Pakistan (29), Ethiopia (30), Ecuador (26), and notably lower than results from a study involving community populations (30). The difference in the knowledge score can be attributed to using different items related to COVID-19 and varying definitions for establishing a “good” level of knowledge. Therefore, it is difficult to directly compare our findings with those of previous studies. However, our study suggests appropriate training is urgently needed among healthcare students in China. Unfortunately, this need appears to be unmet by health facilities and universities, as most of our samples reported a lack of formal training about COVID-19. This represents a concerning gap in the educational preparation of healthcare students in China and an important missed opportunity in the global fight against COVID-19. Future studies should assess these deficiencies in more detail and expand the scope to identify and improve on such training gaps. Faced with a growing shortage of healthcare workers across the major healthcare disciplines, healthcare students are part of the solution to cover and complement this problem (18); however, the absence of knowledge needs urgent attention. Moreover, widespread misconstrued or false beliefs relating to COVID-19 are exacerbating the situation and may pose a potential risk (31). This study highlights that to safely and effectively expand the healthcare workforce, the development of a new curriculum providing additional training to healthcare students that adequately equips them to cover their roles during the COVID-19 crisis should be given priority attention.

In this study, consistent with other studies (31–34), age and level of education were significant influencing factors in the knowledge of students of COVID-19. This suggests that the COVID-19 training program for healthcare students should be focused on younger aged students and low-educated groups, to promote them to form good knowledge. It is worth mentioning that the knowledge of students majoring in oral medicine was higher than others. This could be influenced but the fact that novel coronavirus is mainly transmitted by respiratory droplets and contact (35), and that oral students are educated that close contact with the mouth, nose, or eyes of a person (36) has a high risk of infection through proximity with potentially infected biological fluids (37), especially during an examination, care, and transfer of patients (38). Therefore, compared with other majors, oral medical students had more knowledge about COVID-19. These findings further suggest that health education intervention would be more effective if it targets certain demographic groups (36).

This study revealed optimistic attitudes toward COVID-19 in accordance with other studies from Africa and Italia (39, 40). The optimistic attitude of the Chinese healthcare students could be related to the effectiveness of COVID-19 control in China (41). In addition, the concerted prevention and control efforts implemented across the country also increased the confidence of Chinese people to overcome the epidemic (33), which may in turn have enhanced the confidence of students in winning the battle against the crisis. During the COVID-19 pandemic, many students in healthcare professions helped serve on the front-line and work as volunteers in vaccination programs (42), and knowledge gained in these settings may have also reduced panic and increased confidence in the fight against the virus (42). Our study also found that participating in volunteer activities was a significant positive factor affecting the attitude of medical students. However, there were still students with negative attitudes toward proper management of the crisis. For example from March 2020, with the effective control of the epidemic of COVID-19 in China, colleges and universities began to open and students were required to return to school. This created a sense of fear and outrage (33) as it presented difficulty in practicing social distancing due to dormitory living with four to eight students and increased their risk of exposure to the virus (5). In addition, students, especially those attending clinical training, may have a shortage of PPE, which further exacerbated an already stressful situation (5) and therefore influence their attitudes regarding COVID-19 (11). Multiple organizational and work-related interventions can mitigate these scenarios, such as the improvement of workplace infrastructures, the adoption of correct and shared anti-contagion measures, such as regular PPE supply, and the implementation of resilience training programs, to improve discrimination attitude and knowledge of the students (9).

In the current investigation, despite most students taking precautions to prevent COVID-19, such as not going to crowded places and wearing masks when going outside, the level of practice was still sometimes lower than expected. Of particular concern were the rates of students still going to crowded places without wearing masks and not avoiding handshaking or suspected high-risk areas. This can be linked to the level of the knowledge available among the students, along with unsatisfactory practices cited in other studies, such as the lack of availability (for example, masks and disinfectants) (6, 43), imposing financial costs on participants, ambiguity in instructions, not getting used to new conditions, such as staying home and wearing a mask (44), and exhaustion from existing conditions.

Our study indicated that students who had access to training about COVID-19 report significant knowledge and good practice skills. This reinforces that to increase the understating of the students of the disease and influences their behavior; there is an urgent need to ensure students have access to education and outreach materials. In addition, schools need to strengthen the behaviors of the students by providing access to lifestyle interventions, such as advocating a healthy lifestyle, good hygiene and sanitation practices, strengthening physical exercises, keeping a balanced diet, and ensuring enough sleep (45). In the time of COVID, students also need to be schooled in avoiding contact with respiratory patients and crowded enclosed spaces.

Despite our efforts in reducing the possible shortcomings, this study does have certain limitations. First, the data presented in this study are self-reported and thus may be subject to recall bias. Furthermore, this study was conducted during the COVID-19 pandemic so the results should be taken in this context. Finally, COVID-19 is a respiratory infectious disease, reducing face-to-face communication is one of the measures to prevent and control it (38). During the epidemic period, network investigation has the advantages of high safety, good isolation, and free from time and space restrictions, which can be used as an advantageous supplement to field investigation (31). However, it is difficult to randomize correct samples in a network survey, which may lead to insufficient representatives of samples and the possible limitations of conclusions.

## Conclusions

An increasing effort should be made to improve the knowledge and practices of Chinese healthcare students regarding COVID-19 prevention. This study identified that Chinese healthcare students had positive attitudes toward tackling the disease but were limited in their knowledge about COVID-19 and preventative practices with respect to containing the spread of the disease. Our study also found that students early in their program and those aged under 25 years were significantly less likely to have good knowledge, and thus, education needs to be introduced early into programs. Some participants also continued to attend crowded places without wearing masks and some did not avoid going to suspected high prevalence areas, suggesting undergraduate healthcare profession program providers need to address a gap in KAP toward the preventive measures of COVID-19 at both a personal and professional risk context. The program should also include innovative means of disseminating this information, such as social media, to provide timely and reliable data to students about COVID-19 KAP.

## Data Availability Statement

The original contributions presented in the study are included in the article/supplementary material, further inquiries can be directed to the corresponding author/s.

## Ethics Statement

The studies involving human participants were reviewed and approved by Gansu Provincial Hospital. The patients/participants provided their written informed consent to participate in this study.

## Author Contributions

JZ, YY, YPZ, and JW contributed to conception and design of the study. JZ, YY, XZ, and YYZ organized the database. JW, YY, YPZ, and JD analyzed the data. JZ and YY wrote the first draft of the manuscript. YY, XZ, YYZ, and JD wrote the sections of the manuscript. YPZ, JD, and JW revised the language and content of the paper. JW and JZ provided funding support. All authors contributed to manuscript revision, read, and approved the submitted version.

## Funding

The study was fund by the Science and Technology Plan Project of Chengguan district, Lanzhou, China (2018SHFZ0017), Gansu Science and Technology Plan (Innovation Base and Talent Plan) project (21JR7RA607), National scientific research cultivation project of Gansu Provincial Hospital (19SYPYB-18), and Health industry scientific research project of Gansu Province (GSWSKY-2019-50).

## Conflict of Interest

The authors declare that the research was conducted in the absence of any commercial or financial relationships that could be construed as a potential conflict of interest.

## Publisher's Note

All claims expressed in this article are solely those of the authors and do not necessarily represent those of their affiliated organizations, or those of the publisher, the editors and the reviewers. Any product that may be evaluated in this article, or claim that may be made by its manufacturer, is not guaranteed or endorsed by the publisher.
