# Corrigendum: Knowledge, Attitude, and Practice Survey of COVID-19 Among Healthcare Students During the COVID-19 Outbreak in China: An Online Cross-Sectional Survey

**DOI:** 10.3389/fpubh.2021.829550

**Published:** 2022-01-05

**Authors:** Juxia Zhang, Yuhuan Yin, Judith Dean, Xiaoli Zhang, Yiyin Zhang, Jiancheng Wang, Yinping Zhang

**Affiliations:** ^1^Clinical Educational Department, Gansu Provincial Hospital, Lanzhou, China; ^2^School of Nursing, Gansu University of Chinese Medicine, Lanzhou, China; ^3^School of Public Health, The University of Queensland, Herston, QLD, Australia; ^4^Geriatrics Department, Gansu Provincial Hospital, Lanzhou, China; ^5^School of Nursing, Xi'an Jiaotong University Health Science Center, Xi'an, China

**Keywords:** knowledge, attitude, practice, COVID-19, China, health care

**Incorrect Affiliation**

In the published article, there was an error in affiliation “1,2,3,4,5”. Instead of “1 School of Nursing, Xi'an Jiaotong University Health Science Center, Xi'an, China; 2 Clinical Educational Department, Gansu Provincial Hospital, Lanzhou, China; 3 School of Nursing, Gansu University of Chinese Medicine, Lanzhou, China; 4 School of Public Health, The University of Queensland, Herston, QLD, Australia; 5 Elder Office, Gansu Office, Gansu Provincial Hospital, Lanzhou,”, it should be “1 Clinical Educational Department, Gansu Provincial Hospital, Lanzhou, China; 2 School of Nursing, Gansu University of Chinese Medicine, Lanzhou, China; 3 School of Public Health, The University of Queensland, Herston, QLD, Australia; 4 Geriatrics Department, Gansu Provincial Hospital, Lanzhou, China; 5 School of Nursing, Xi'an Jiaotong University Health Science Center, Xi'an, China”.

The authors apologize for this error and state that this does not change the scientific conclusions of the article in any way. The original article has been updated.

## Publisher's Note

All claims expressed in this article are solely those of the authors and do not necessarily represent those of their affiliated organizations, or those of the publisher, the editors and the reviewers. Any product that may be evaluated in this article, or claim that may be made by its manufacturer, is not guaranteed or endorsed by the publisher.

